# Unraveling Morphophysiological and Biochemical Responses of *Triticum aestivum* L. to Extreme pH: Coordinated Actions of Antioxidant Defense and Glyoxalase Systems

**DOI:** 10.3390/plants8010024

**Published:** 2019-01-18

**Authors:** M. H. M. Borhannuddin Bhuyan, Mirza Hasanuzzaman, Jubayer Al Mahmud, Md. Shahadat Hossain, Tasnim Farha Bhuiyan, Masayuki Fujita

**Affiliations:** 1 Laboratory of Plant Stress Response, Department of Applied Biological Sciences, Faculty of Agriculture, Kagawa University, Miki-cho, Kita-gun, Kagawa 761-0795, Japan; razon_sau@yahoo.com (M.H.M.B.B.); shahadatsau24@gmail.com (M.S.H.); 2 Citrus Research Station, Bangladesh Agricultural Research Institute, Jaintapur, Sylhet 3156, Bangladesh; 3 Department of Agronomy, Sher-e-Bangla Agricultural University, Dhaka 1207, Bangladesh; mhzsauag@yahoo.com; 4 Department of Agroforestry and Environmental Science, Sher-e-Bangla Agricultural University, Dhaka 1207, Bangladesh; jamahmud_bd@yahoo.com; 5 Department of Agricultural Botany, Sher-e-Bangla Agricultural University, Dhaka 1207, Bangladesh; farhatasnim28@gmail.com

**Keywords:** acidity, alkalinity, antioxidant defense, methylglyoxal, phytotoxicity, reactive oxygen species

## Abstract

Soil pH, either low (acidity) or high (alkalinity), is one of the major constraints that affect many biochemical and biological processes within the cell. The present study was carried out to understand the oxidative damage and antioxidant defense in wheat (*Triticum aestivum* L. cv. BARI Gom-25) grown under different pH regimes. Eight-day-old seedlings were exposed to growing media with different pH levels (4.0, 5.5, 7.0, and 8.5). Seedlings grown in pH 4.0 and in pH 8.5 showed reductions in biomass, water, and chlorophyll contents; whereas plants grown at pH 7.0 (neutral) exhibited a better performance. Extremely acidic (pH 4.0) and/or strongly alkaline (pH 8.5)-stress also increased oxidative damage in wheat by excess reactive oxygen species (ROS) generation and methylglyoxal (MG) production, which increased lipid peroxidation and disrupted the redox state. In contrary, the lowest oxidative damage was observed at a neutral condition, followed by a strong acidic condition (pH 5.5), which was mainly attributed to the better performance of the antioxidant defense and glyoxalase systems. Interestingly, seedlings grown at pH 5.5 showed a significant increase in morphophysiological attributes compared with extreme acidic (pH 4.0)- and strong alkaline (pH 8.5)-stress treatments, which indicates the tolerance of wheat to the acidic condition.

## 1. Introduction

Abiotic stresses hamper crop production and challenge farmers to grow food for the enormous world community, which will reach 10.9 billion by 2050 [1]. Among the abiotic stressors, extreme pH, both acidity and alkalinity, covers about 60 percent of the global land surface, with spatial variability [2]. Soil pH is the indicator used to describe the acidity or alkalinity of the soils. The Soil Survey Division Staff, USDA [3], proposed nine soil classes based on pH: ultra acidic (pH < 3.5), extremely acidic (3.5–4.4), very strongly acidic (4.5–5.0), strongly acidic (5.1–5.5), moderately acidic (5.6–6.0), slightly acidic (6.1–6.5), neutral (6.6–7.3), slightly alkaline (7.4–7.8), moderately alkaline (7.9–8.4), strongly alkaline (8.5–9.0), and very strongly alkaline (>9.0). From an agricultural point of view, pH between 6.0 and 7.3 is best. However, some plants prefer pH 4.0 to 5.5 (e.g., potato, rye, blueberry, cranberry), some grow better at pH 5.5 to 6.5 (e.g., barley, rice, carrot, eggplant, cauliflower), some prefer pH 6.0 to 7.0 (e.g., maize, cabbage, mustard, kohlrabi), and some crops (garlic, pepper, winter squash etc.) can grow in a wide range of soil pH (5.0–7.5) [4]. Geographic, geological, meteorological biological, and anthropogenic factors are responsible for developing either acidity or alkalinity [2], thus hampering the productivity of crops by exerting stresses [5].

Plant cells need a range of pH 7.0–7.5 in cytoplasm to maintain a normal physiological function [6]. The pH of the external growing media exerts a negative impact on the cellular pH. Besides, the enzymes that accomplish the normal physiological activities are also pH dependent. Reports suggest that a decrease of 1 in the pH of the external growing media reduces the cytoplasmic pH by around 0.1 units [7]. On the other hand, when the pH of the external growing media increases, it causes the precipitation of phosphorus and metal ions, increases the absorption of inorganic anions, and disrupts the ion balance [8]. Therefore, both acidic and alkaline pH of growing media alters pH homeostasis in the tissues. To combat extreme pH stress-induced cellular pH alteration, proton pumps play an important role in the adaptation to acidic or alkaline stress by mediating proton efflux and influx [9,10]. However, their effectiveness to balance the cytoplasmic pH reduces gradually [6], and eventually disrupts many cellular activities, including enzyme activities. Moreover, excess energy dissipation may raise toxic reactive oxygen species (ROS) [11,12], including singlet oxygen (^1^O_2_), superoxide anion (O_2_^−^), hydrogen peroxide (H_2_O_2_), and hydroxyl radical (OH). Reports suggest that a reduction of 0.5 units in pH tends to increase the ROS accumulation by many folds in *Hordium vulgare* L. [11] and *Pinus sylvestris* L. [13], whereas the increase in pH also generates oxidative stress in *Malus* spp rootstocks [12]. 

These toxic ROS can oxidize important cellular ultrastructures, leading to oxidative damage and the destruction of cellular organelles [14]. Fortunately, plant cells are coupled with the antioxidative system to minimize the oxidative stress comprised of the non-enzymes (ascorbic acid, AsA; glutathione, GSH; phenolic compounds; alkaloids; α-tocopherol; non-protein amino acids) and enzymes (superoxide dismutase, SOD; catalase, CAT; ascorbate peroxidase, APX; glutathione reductase, GR; monodehydroascorbate reductase, MDHAR; dehydroascorbate reductase, DHAR; glutathione peroxidase, GPX; and glutathione *S*-transferase, GST), which work coordinately to detoxify the toxic ROS [15,16,17]. Another cytotoxic compound, the glycolysis byproduct methylglyoxal (MG), was also reported to be overproduced under abiotic stress [16], which can damage cells by creating oxidative stress. The MG detoxification system comprised of glyoxalase I (Gly I) and glyoxalase II (Gly II) enzymes helps to detoxify MG [18]. Reports suggest that the metabolomic modification of the antioxidant and glyoxalase system can improve abiotic stress tolerance [14,17,19,20,21]. However, to mechanize the antioxidant defense and glyoxalase system under extreme pH, the first requirement is to elucidate different attributes that are disturbed, and which can further be targeted to modulate using phytoprotectants and/or genetic manipulation. A few reports have demonstrated the involvement of oxidative damage generation by acidity or alkalinity, yet a comparative physiological study is unavailable. Moreover, there is no information available on the extreme pH-induced regulation of the antioxidative and glyoxalase systems.

Wheat (*Triticum aestivum* L.) ranks first among the food grain crops and its productivity is negatively affected by acidic or alkaline conditions. Therefore, we examined the growth and biomass, water status, photosynthetic pigments, oxidative damage, and performance of the antioxidant and glyoxalase systems of wheat under extreme pH-stress during the early seedling stage. To the best of our knowledge, this is the first report to elucidate the negative impact of extreme-pH stress on wheat seedlings, in which the co-ordinated actions of the antioxidant and glyoxalase systems have been investigated.

## 2. Results

### 2.1. Growth and Biomass Accumulation 

**Figure 1:**
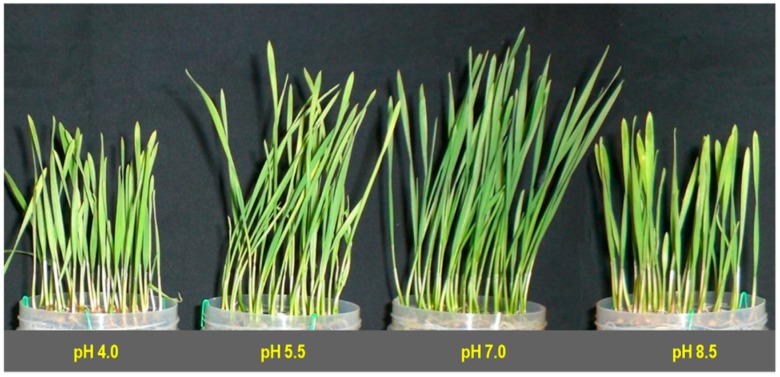
Visual images of morphological differences among wheat (*Triticum aestivum* L. cv. BARI Gom-25) seedlings grown under different pHs.

Extreme pH of the growing media influenced the growth and biomass accumulation of the wheat seedlings. Upon exposure to extremely acidic (pH 4.0)-stress, the plant height was reduced by 33% compared with the control, where a 14% reduction in shoot length resulted in strongly acidic (pH 5.5)-stress. Similarly, strongly alkaline (pH 8.5)-stress reduced the shoot height by 31% compared to the control (Table 1; Figure 1). 

The root length was also influenced by extreme pH-stress. Compared to the control, upon exposure to acidic pH-stress (pH 4.0 and pH 5.5), root length reduced by 18% and 5% respectively, whereas an 18% reduction in root length was observed in seedlings grown in strongly alkaline (pH 8.5)-stress compared with the control (Table 1). 

Extreme pH-stress again hampered the biomass accumulation of wheat seedlings. The fresh weight of shoots and roots was reduced by 12 and 27%, respectively, upon exposure to an extremely acidic (pH 4.0)-stress condition, while a strongly acidic (pH 5.5)-stress condition reduced the shoot and root fresh weight by 5 and 12%, respectively. On the other hand, strongly alkaline (pH 8.5)-stress reduced the shoot and root fresh weight by 17 and 26%, respectively (Table 1). 

The dry weight of shoots and roots declined by 7 and 35%, respectively, upon exposure to extremely acidic (pH 4.0)-stress, while strongly acidic (pH 5.5)-stress did not reduce the shoot dry weight but reduced the root dry weight by 6%. Similarly, strongly alkaline (pH 8.5)-stress reduced the shoot and root dry weight by 6 and 32%, respectively (Table 1).

### 2.2. Relative Water and Proline Content

Extreme pH-stress alters the leaf RWC of the wheat seedlings. Compared to the control, leaf RWC was reduced by 13 and 6% at an acidic-stress (pH 4.0 and pH 5.5, respectively) condition; while a 14% reduction in leaf RWC was observed for the alkaline-stressed (pH 8.5) seedlings (Table 1). 

The contents of Pro were boosted upon exposure to an extreme pH condition in wheat seedlings, and compared to the control, a 19- and 4-fold increase in Pro content was found in acid-stressed seedlings (pH 4.0 and pH 5.5, respectively). Alkaline-stress (pH 8.5) also gave rise to a 22-fold increase in Pro content compared to the control (Table 1).

### 2.3. Oxidative Stress Markers

**Figure 2:**
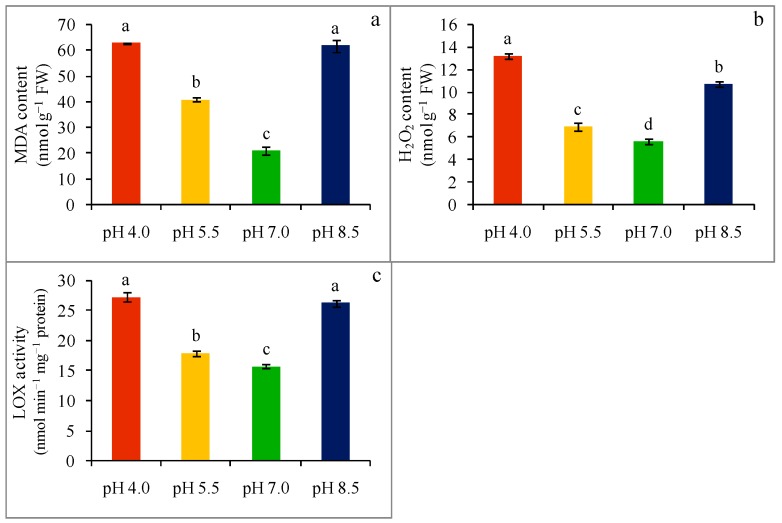
Malondialdehyde (MDA) content (**a**), H_2_O_2_ content (**b**), and LOX activity (**c**), of wheat leaves under different levels of pH. Mean (±SD) was computed from three replications of each treatment. Bars with dissimilar letters are significantly different at *p* ≤ 0.05 from Fisher’s LSD test.

Malondialdehyde—a stress indicator produced from lipid peroxidation in leaf tissues, was determined and is illustrated in (Figure 2a). Compared to control (pH 7.0) seedlings, MDA content increased by 199 and 194% in pH 4.0 and pH 8.5 exposed seedlings, respectively. However, seedlings grown on pH 5.5 showed lower increases in MDA content (95%) compared with control seedlings.

In line with MDA, a remarkable rise in H_2_O_2_ content was noticed in leaf tissue upon exposure to varying rhizosphere pH (Figure 2b). Compared with the control, a sharp increase in H_2_O_2_ content (134 and 90%) was observed at both an extremely acidic (pH 4.0) and strongly alkaline (pH 8.5) pH. However, seedlings grown under a strongly acidic (pH 5.5)-stress condition were found with a slightly increased H_2_O_2_ content (30%) compared to the control.

Similarly, varying the rhizosphere pH increased the activity of lipoxygenase (LOX) drastically. Upon exposure to extreme pH, LOX activity increased by 73 and 67% (compared with control) in extremely acidic (pH 4.0)- and strongly alkaline (pH 8.5)-stressed seedlings, respectively. However, compared with the control, seedlings grown under strongly acidic (pH 5.5)-stress were found to exhibit a slight increase (23%) in LOX activity (Figure 2c). 

### 2.4. Photosynthetic Pigment Contents

**Figure 3:**
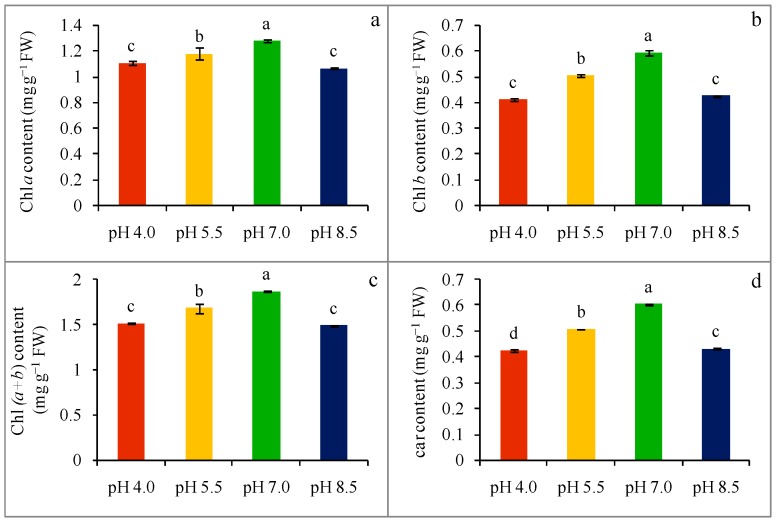
Chlorophyll a (**a**), chl b (**b**), total chl (a + b) (**c**), and car (**d**) contents of wheat leaves under different levels of pH. Mean (±SD) was computed from three replications of each treatment. Bars with dissimilar letters are significantly different at *p* ≤ 0.05 from Fisher’s LSD test.

Extreme rhizosphere pH destroyed chl in leaf tissues. Chlorophyll a content was decreased in acid-stressed wheat seedlings in a dose-dependent manner by 13 and 8% at pH 4.0 and pH 5.5, respectively (Figure 3a), while alkaline rhizospheric pH (8.5) also diminished chl a content by 17% compared with the control. Extreme pH-stress also altered chl b content in a similar fashion. Compared to the control, a 31 and 15% decrease in chl b content was found in acidic conditions of pH 4.0 and pH 5.5, respectively, while compared to the control, a 28% reduction in chl b content was found in an alkaline (pH 8.5) condition (Figure 3b). Extreme pH-stress once more reduced chl (a + b) content in comparison with the control seedlings. Hence, the chl (a + b) values were decreased by 19 and 10% compared with the control in acid-stress, pH 4.0 and pH 5.5 treated seedlings, respectively (Figure 3c), whereas compared to the control, a 20% decrease in chl (a + b) content was found in alkaline (pH 8.5) treated seedlings. Another photosynthetic leaf pigment, car, was also reduced by the negative effect of extreme pH, and compared to the control, a 30 and 16% reduction was observed in an acidic (pH 4.0 and pH 5.5, respectively) condition, while a 28% reduction in car content was found at in an alkaline (pH 8.5) condition (Figure 3d). The chlorotic symptoms were also visible in the leaves in seedlings exposed to extreme pH (Figure 1).

### 2.5. Nonenzymatic Antioxidant Content 

**Figure 4:**
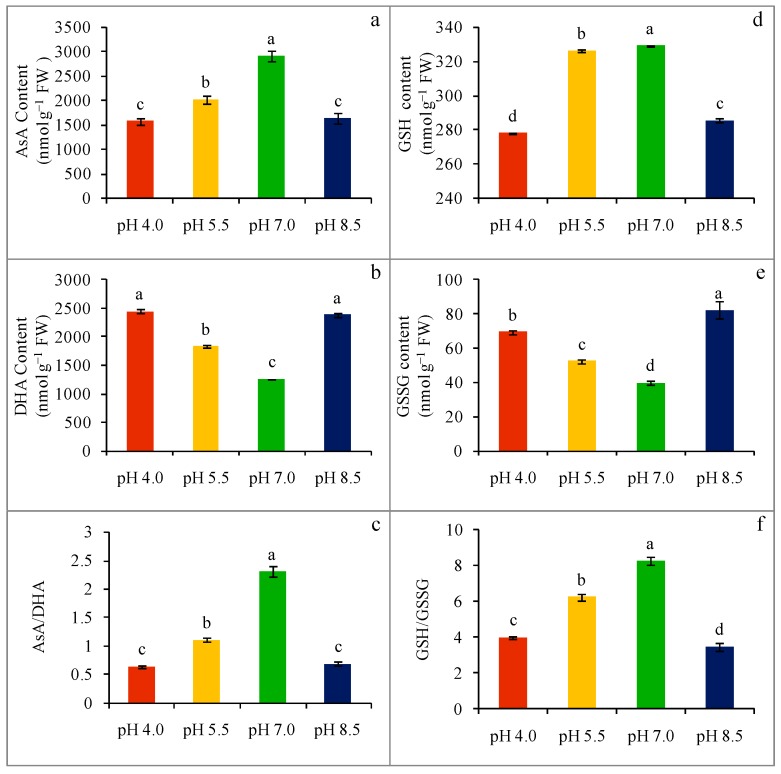
AsA (**a**) and DHA (**b**) contents, AsA/DHA ratio (**c**), GSH (**d**) and GSSG (**e**) contents, and GSH/GSSG ratio (**f**) of wheat leaves under different levels of pH. Mean (±SD) was computed from three replications of each treatment. Bars with dissimilar letters are significantly different at *p* ≤ 0.05 from Fisher’s LSD test.

The non-enzymatic antioxidant contents of wheat seedlings were affected greatly upon exposure to extreme pH. A sharp decrease in AsA content was noticed in respect of acidity-stress, which was 46 and 31% in seedlings subjected to pH 4.0 and pH 5.5, respectively, compared to the control (Figure 4a), while strongly alkaline-stressed (pH 8.5) seedlings displayed a 76% decrease in AsA content compared to the control seedlings. A noticeable increase in DHA content (94 and 45 and 89% in pH 4.0, pH 5.5, and pH 8.5, respectively) was observed compared to the control (Figure 4b). Extreme pH stress lessened the AsA/DHA ratio in wheat seedlings. Compared to the control, a 72 and 52% decrease in the AsA/DHA ratio was observed in pH 4.0 and pH 5.5 acidity-stresses, respectively, whereas the AsA/DHA ratio was decreased by 70% in alkaline-stressed seedlings compared with the control (Figure 4c).

Glutathione content was also altered by the effect of extreme pH. It was observed that 16% of the decrease in GSH content occurred upon exposure to an extreme acidic condition, whereas a 15% decrease in GSH content was found in a strong alkaline-stress (pH 8.5) condition compared with the control (Figure 4d). However, a strong acidic condition did not alter the GSH content (Figure 4d). On the other hand, GSSG content was increased regarding extreme pH-stress. The GSSG content increased by 74 and 31% in acidity-stress (pH 4.0 and pH 5.5, respectively), while strong alkaline-stress (pH 8.5) increased the GSSG content by 106% compared to the control (Figure 4e). The ratio of GSH and GSSG was also changed due to extreme pH-stress. In response to acidity-stress, a 52 and 25% decrease was observed in the GSH/GSSG ratio under pH 4.0 and pH 5.5, respectively. Whereas, a 58% decrease in the GSH/GSSG ratio was observed in strongly alkaline (pH 8.5)-stress (Figure 4f). 

### 2.6. Enzyme Activity

**Figure 5:**
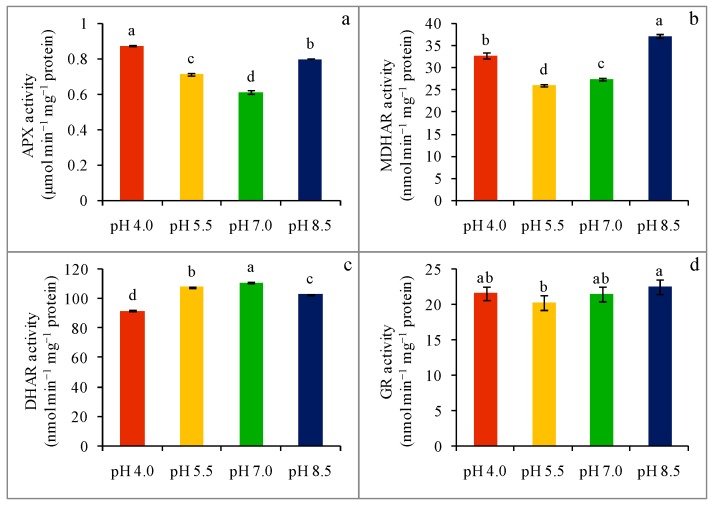
Activities of APX (**a**), MDHAR (**b**), DHAR (**c**), and GR (**d**) of wheat leaves under different levels of pH. Mean (±SD) was computed from three replications of each treatment. Bars with dissimilar letters are significantly different at *p* ≤ 0.05 from Fisher’s LSD test.

Compared to the control, acidity-stressed (pH 4.0 and pH 5.5) wheat seedlings were found with 43 and 17% upregulated APX activity, whereas alkaline-stressed (pH 8.5) seedlings also showed an upregulation of APX activity by 30% (Figure 5a). A strongly acidic (pH 5.5) condition reduced the activity of the MDHAR enzyme by 15% in comparison to untreated seedlings, while an extremely acidic (pH 4.0) condition and strongly alkaline condition (pH 8.5) favored the upregulation of MDHAR activity by 19 and 35%, respectively, compared with the control (Figure 5b). Unlike MDHAR, the DHAR enzyme activity was down-regulated by 17 and 3% in acidity-stress (pH 4.0 and pH 5.5, respectively) compared with the control seedlings (Figure 5c), whereas DHAR activity reduced by 7% in alkaline-stressed (pH 8.5) seedlings. On the other hand, in contrast to the control seedlings, extreme pH exposure did not alter GR activity significantly (Figure 5d).

**Figure 6:**
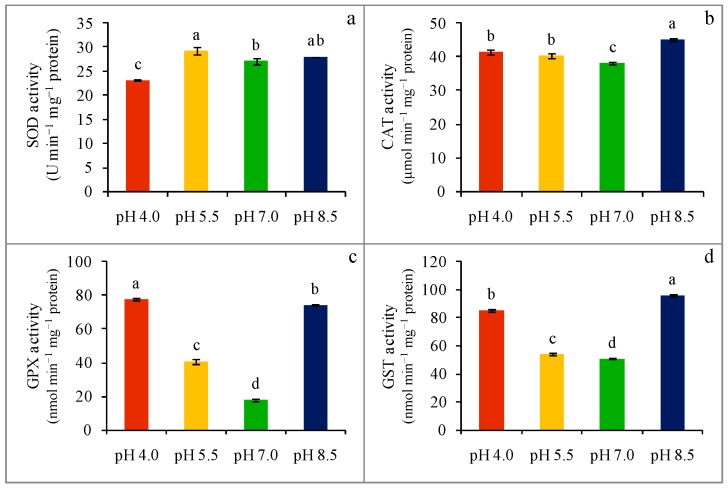
Activities of SOD (**a**), CAT (**b**) GPX (**c**), and GST (**d**) of wheat leaves under different levels of pH. Mean (±SD) was computed from three replications of each treatment. Bars with dissimilar letters are significantly different at *p* ≤ 0.05 from Fisher’s LSD test.

The activity of SOD was down-regulated by 15% in response to extremely acidic-stress pH 4.0 (Figure 6a). On the other hand, SOD activity remained statistically indifferent in strongly acidic (pH 5.5)- and strongly alkaline (pH 8.5)-stressed seedlings (Figure 6c). The activity of CAT was also up-regulated in response to extreme pH stress (Figure 6b). Catalase activity increased by 9 and 6% in pH 4.0 and pH 5.5 acidic-stressed wheat seedlings, respectively, whereas, compared to the control, strongly alkaline (pH 8.5)-stressed seedlings were found with an 18% upregulation of CAT activity (Figure 6b). A considerable increase in GPX activity was observed in wheat seedlings upon exposure to extreme pH-stress, which was 332 and 128% higher in acidity-stressed (pH 4.0 and pH 5.5, respectively) seedlings, while a 313% increase in GPX activity was found in strongly alkaline (pH 8.5)-stressed seedlings (Figure 6c). The GST activity was also upregulated at any level of extreme pH-stress. Compared to the control, GST activity was increased by 68, 6, and 89% in extreme pH-stressed (acidic pH 4.0 and pH 5.5, and alkaline pH 8.5, respectively) seedlings (Figure 6d). 

### 2.7. Glyoxalase Enzymes Activity and Methylglyoxal Content

**Figure 7:**
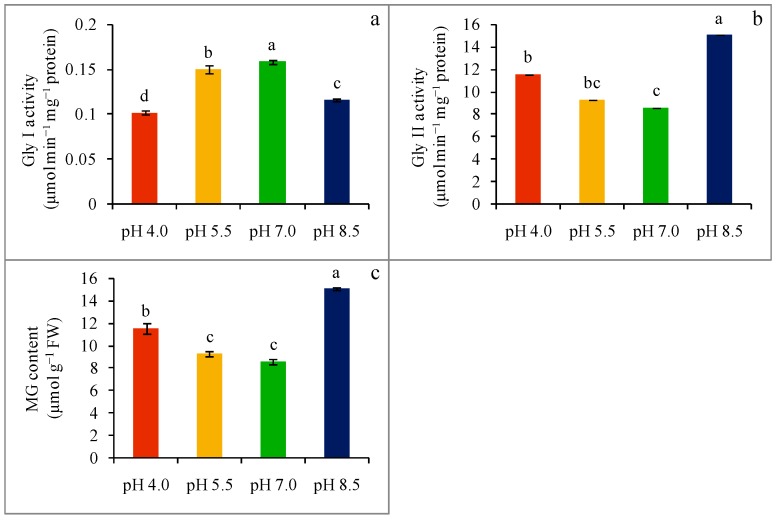
Activities of Gly I (**a**) and Gly II (**b**) and MG contents (**c**) of wheat leaves under different levels of pH. Mean (±SD) was computed from three replications of each treatment. Bars with dissimilar letters are significantly different at p ≤ 0.05 from Fisher’s LSD test.

Glyoxalase system comprised of enzyme Gly I and Gly II activity was distorted due to the extreme pH of the growing media, and as a result, MG detoxification was also hampered. The acidity (pH 4.0 and pH 5.5) of growing media decreased Gly I activity by 36 and 5%, respectively (Figure 7a), whereas a 27% reduction in Gly I activity was attributed to an alkaline (pH 8.5) growing condition compared to the control. Meanwhile, with respect to the control, Gly II activity was reduced by 12, 5, and 27% in the extreme pH conditions (pH 4.0, pH 5.5, and pH 8.5, respectively) of the growing media (Figure 7b).

Because of the down-regulation of these two vital enzymes, MG content increased notably. Compared with the control seedlings, MG content was increased by 35 and 8% in acidity-stressed (pH 4.0 and pH 5.5, respectively) seedlings, while a sharp increase (78%) in MG content was observed when the seedlings were exposed to a strong alkaline condition (Figure 7c).

## 3. Discussion

Crop productivity can be hampered by the extreme (both low and high) pH of the growing media. Both acid and alkaline soils are one of the most important limitations to agricultural production worldwide [22]. It has been reported that alkali stress involves the same stress factors as salt stress, with the added involvement of a high pH, and the injurious effects on plants are more severe than salt stress, where root growth reduction is much greater than that of shoots [23]. A similar growth reduction was also reported in *Solanum lycopersicum* [24]. On the contrary, due to H^+^ rhizooxicity, acidity stress inhibits root growth and leads to shallow root systems, hampering water and nutrient uptake [25]. Hence, both acidity and alkalinity increase the risk of drought stress.

In the present study, we found the negative effects of extreme pH, including both acidity- and alkalinity-stress, on the morphophysiological attributes of wheat seedlings. Exposure to both acidity and alkalinity, a significant decrease in the seedling growth in terms of seedling height, root length, fresh weight, and dry weight of both shoots and roots was observed, which corroborates with previous studies [23,24,25,26]. A significant reduction in root elongation is reported under a low pH (pH 4.0) due to the higher H^+^ toxicity in extremely acidic soil of crop plants such as alfalfa, wheat, spinach, common bean, barley etc. [23,24]. This reduction in root growth might be due to the decrease of root cell division and enlargement [22]. Similarly, Silva et al. [26] reported the reduction in root length and both shoot and root biomass under alkaline stress (>pH 7.0) due to the higher pH and metal(loids) toxicity. 

As a sessile organism, plants cannot avoid the occurrence of environmental stress and one of its obvious consequences, i.e., the overgeneration of ROS (O_2_^−^, H_2_O_2_, OH^−^). In line with other abiotic stress factors, under extreme pH, including both acidic and alkaline-stress, we observed elevated lipid peroxidation (measured as MDA), H_2_O_2_ overgeneration, and increased LOX activity in the leaf tissue of wheat seedlings, which is consistent with other studies [11,13,27,28]. Alkali stress induced greater cellular structural damage and higher lipid peroxidation was found in tomato plants [27]. Similarly, acidic stress (pH 4.5)-induced lipid peroxidation (higher MDA content) was also observed in *H. vulgare* seedlings [11]. In parallel, rising ROS content and lipid peroxidation was also found in *P. sylvestris* and *Lotus corniculatus, Oryza sativa*, and citrus following exposure to acidity [13,28,29,30]. 

Extreme pH-induced ROS production might also be involved in the breakdown of photosynthetic pigments, which was evident in the present study. We found the lowering of photosynthetic pigments content viz. chl *a,* chl *b*, chl (*a + b*), and car, which corroborates with the previous studies. Yet, ionic imbalance and a disturbance in pH homeostasis within plant tissue under alkalinity-stress cause the precipitation of metal ions, consequently responsible for photosynthetic pigments breakdown and subsequent leaf chlorosis, a reduced photosynthetic rate, and stunted growth [9]. Similarly, a decreased photochemical efficiency, and chl and car content were reported under a higher pH (alkali stress) in *Cucumis sativus*, *Medicago sativa*, and *O. sativa* [31]. Thus, this reduction in photosynthesis leads to lower biomass accumulation. On the other hand, Long et al. [30] found that a low pH (2.5) induced the alteration of chl pigments contents, which further reduced the photosynthetic capacity, as well as CO_2_ assimilation, in citrus; but they did not find any changes regarding the photosynthetic efficiency at pH ≥ 2.5. However, a differential response to the same stress may occur due to the genetic makeup, which indicates a higher tolerance of citrus to acidity stress.

We found reduced RWC in the leaf tissue of wheat seedlings due to extreme pH, both acidity and alkalinity, which might be attributed to the reduced root length due to the alteration in growing media pH, which subsequently caused water unavailability in the growing shoot, and induced artificial drought to the plants. Many of the research reports suggest that abiotic stresses are responsible for reducing the RWC in the plants [14,16,17,19]. Yang et al. [9] reported that a higher rhizosphere pH caused a lowered water content in *T. aestivum* seedlings; likewise, reduction was also found in *Helianthus annus*, *Aneurolepidium chinense*, and *T. aestivum* seedlings [9]. Similarly, low pH-induced root damage and reduced RWC were also observed under acidity stress [29,30]. 

Proline is a low molecular weighted amino acid, and is well-known as an osmoregulator and ROS scavenger [15]. The adjacent relation between Pro accumulation and dehydration tolerance is the basic strategy to avoid a detrimental effect on many major physiological processes, such as leaf expansion, the retention of cell osmotic potential, stomatal conductance, and photosynthesis. Therefore, the higher Pro accumulation in our study, due to acidity- or alkalinity-stress, might be because of the increase in Pro biosynthesis, together with a decrease in its oxidation [32]. Abiotic stress-induced overaccumulation of Pro was reported under various abiotic stressors in many previous studies, which further gave protection to stress-induced dehydration and oxidative damage [14,16,17,19]. Therefore, the elevated Pro content in the present study under both acidic and alkaline stress might have given protection against oxidative injury, as well as maintained the water balance. Extreme pH exposure reduced the photosynthetic pigments; hence, the energy converted from the sunlight could not be consumed. This excess energy activates the triplet oxygen (^3^O_2_) to singlet oxygen (^1^O_2_) and subsequently produces reactive O_2_^−^ [33]. The superoxide dismutase enzyme is the first line defense that dismutases O_2_^–^ and converts it to H_2_O_2_ [34]. In the present study, SOD activity slightly increased under mild acidic- and strong alkaline-stress. It was reported that SOD activity gradually but significantly increased in plants under acidity [13], but in our study, SOD activity further decreased under extreme acidity stress. This might be due to the overproduction of O_2_^−^ and H_2_O_2_ at extreme acidity stress; hence the enzyme molecules might be unable to release their product due to the higher concentration of H_2_O_2_ at the releasing pool. On the other hand, higher CAT activity is vital to reduce the H_2_O_2_ content [16]. Although we observed a remarkable upregulation of CAT activity under extreme pH-stress, the H_2_O_2_ content was not reduced; hence it can be mentioned that the upregulation of CAT activity was not great enough to scavenge the overproduced H_2_O_2_, which indicates the necessity of the AsA-GSH pathway in scavenging overgenerated H_2_O_2_. At the AsA-GSH pool, we observed a decrease in AsA content and increased APX activity during extreme pH-stress, which are probably due to scavenging H_2_O_2_. This reduction of AsA content also resulted from reduced DHAR activities, which increased the DHA content. Although the MDHAR activity increased in both strongly acidic and alkaline conditions, the AsA/DHA ratio could not be maintained. Alternatively, H_2_O_2_ scavenging lowered and seedlings suffer from a severed oxidative load. The result of our study is in line with previous studies [12,35]. The activity of the ROS detoxification enzymes was significantly increased in plants under acidified [35] and alkaline media [8], indicating that the anti-oxidative defense mechanism is directly involved in the response of seedlings to extreme pH-stress. Previous reports have shown that low pH stress also decreases the activities of SOD and CAT in *Cucumis sativus* [36]. Decreased SOD and CAT activities indicate that the ability to scavenge O_2_^−^ and H_2_O_2_ is weakened by low pH-stress, which may result in ROS-induced damage, including lipid peroxidation in membranes [37]. Generally, an appropriate intracellular balance between ROS generation and scavenging exists in cells. The redox homeostasis requires the efficient coordination of an array of antioxidants that can scavenge ROS and protect cells from oxidative damages. Beside AsA, the S-containing potent antioxidant—GSH, also boosts ROS scavenging, together with other enzymes GPX/GST, while GSH also contributes to xenobiotics detoxification coordinating with the enzyme GST. In our study, strong acidity- and alkalinity-stress reduced the content of GSH, and consequently increased the GSSG content. Although the DHAR activity decreased, at the same time, the GR activity did not noticeably increase. Therefore, an over generated GSSG content in the stress-affected seedlings was observed. On the other hand, the GSH costing two enzymes GPX and GST activities increased in extreme pH-stress; as a result, the GSH content decreased, which corroborates with previous studies [16,19]. It was reported that GPX plays a vital role in scavenging overgenerated H_2_O_2_ and lipid hydroperoxides with the help of GSH, which prevents ROS-induced oxidative damage and confers stress tolerance [15,16]. On the other hand, increased GST activity is also important for ROS detoxification and abiotic stress tolerance [16,19]. Therefore, the alteration in the antioxidant activity (both enzymatic and nonenzymatic) in wheat seedlings is the defense mechanism employed to avoid oxidative damage [38] and boost redox homeostasis to provide tolerance [19,39]. However, a differential response to the same stress might be due to the genetic makeup of a species, where the tolerant individuals may show a higher antioxidative capacity, and might get more protection against severe oxidative damage [40]. 

Another potent cytotoxic glycolytic byproduct, MG, is over-produced at an abiotic stress condition [41]. The overproduction of MG hampered the normal morphophysiological processes like growth, root elongation, and the photosynthetic process in *Arabidopsis* [41]. Furthermore, a higher accumulation of MG results in the inhibition of various physiological processes, like the disruption of the antioxidant defense, and other metabolic dysfunctions, like the inhibition of protein biosynthesis and functions, and nucleic acid biosynthesis, as well as the glycation of proteins [42]. In this process, Gly I and Gly II, two vital enzymes, work together with GSH to eliminate MG; hence, this MG detoxification pathway is also termed the GSH-dependent glyoxalase pathway [43]. In the present study, we found a higher MG content under extreme pH-stresses, which corroborates with the decreased activities of Gly I and Gly II enzymes. This result is coherent with previous research reports, which suggested that ROS enhanced MG production, while MG induced ROS production, and vice versa [41]. Therefore, the reduction of the activity of any of these two vital enzymes might be attributed to severe oxidative stress [17,19]. Modulation of the MG detoxification system is also reported as an effective way to reduce the overproduced MG under various abiotic stresses [18,43]. 

Therefore, the alteration of the wheat seedlings physiology due to extreme pH can be anticipated based on the present study as the root growth inhibition followed by the reduction in water uptake and dehydration stress, subsequent ROS and MG generation, and destruction of photosynthetic pigments due to oxidative damage. Meanwhile the antioxidative and glyoxalase systems were activated to detoxify the overgenerated ROS and MG, but failed after a certain extent. Consequently, the plant died. However, interestingly, the seedlings grown under a strong acidic condition (pH 5.5) performed better compared with the seedlings grown under an extreme acidic (pH 4.0) and strong alkaline (pH 8.5) condition, which indicates that wheat can tolerate a strong acidic (pH 5.5) condition by regulating the antioxidant and glyoxalase system. 

## 4. Materials and Methods

### 4.1. Plant Materials and Stress Treatments

Wheat (*Triticum aestivum* L. cv. BARI Gom-25) seeds were manually sorted by hand for any dirt, debris, and small-sized or dead seeds. Separated quality seeds were then surface sterilized with 1% sodium hypochlorite for 10 min and washed a number of times with deionized water. Plastic vessels of an 8 cm diameter and volume of 25 mL were used for growing the seedlings. The vessels were prepared with a plastic net on top of them and 210 mL of distilled water, and 45 seeds were planted in each vessel on top of the net. The vessels were then incubated for 40 h. After that, the vessels were transferred to the growth chamber after keeping the 25 best seedlings and grown under managed conditions of light 350 µmoL photon m^−1^ s^−2^, temperature 25 ± 2 °C, and relative humidity 65–70% in a cultivation chamber for 6-d. During this tenure, 5000-fold diluted Hyponex solution (Hyponex, Japan) was supplied as a nutrient to the seedlings controlling the pH value at 6.8–7.0 and changed every alternate day. At 8-d, the seedlings were grouped in 4 and exposed to a nutrient solution of three different levels of acidic and alkaline pH viz. 4.0, 5.5, and 8.5, along with a neutral pH as a control, for 72 h. The pH of the nutrient solution was checked daily and adjusted to the desirable pH value again. Hence, the experiment consisted of four treatments viz. (a) extremely acidic (pH 4.0), (b) strongly acidic (pH 5.5), (c) neutral (pH 7.0) or control, and (d) strongly alkaline (pH 8.5) fitted in a completely randomized design (CRD) with three repetitions. After 72 h of extreme pH stress, leaves from different treatments were harvested separately and data were collected following standard methodologies described later. The experimental outline has been presented in Figure S1.

### 4.2. Growth and Biomass Accumulation

The length of the seedlings was recorded after 72 h of stress treatment. The height was measured from the base to the leaf tip of 10 randomly selected plants and the mean value was expressed in cm for shoot length. Similarly, the root length also measured for those selected plants from the base to the root tip of the longest root and the average value was expressed in cm as root length.

Ten randomly selected fresh seedlings from each treatment were incised at the joint of root and shoot and weighed separately, recorded, and considered as the fresh weight of root and shoot. Dry weights were found after drying the seedlings at 80 °C in an oven for 48 h. Both the fresh weight and dry weight of roots and shoots were expressed as g seedling^−1^.

### 4.3. Determination of Stress Markers

Malondialdehyde (MDA) and H_2_O_2_, two vital stress markers, were determined according to Heath and Packer [44] and Yu et al. [45], respectively. Malondialdehyde was measured as thiobarbituric acid-reacting substances (TBARS) after extracting a 0.5 g fresh leaf sample with 5% trichloroacetic acid (TCA) and centrifuging it at 11,500× *g*. Resultant supernatant (1 mL) was mixed with 4 mL of 0.5% TBA (in 20% TCA) and incubated at 95 °C for 30 min, before being quickly cooled in ice to terminate the reaction, and the absorbance was read at 532 and 600 nm, calculated using the extinction coefficient (155 mM^−1^cm^−1^) and expressed as nmol g^−1^FW [44]. 

The amount of H_2_O_2_ accumulation was determined using 5.5 mM TiCl_4_ (in 20% H_2_SO_4_) after extracting the 0.5 g plant sample with 3 mL of potassium-phosphate (K-P) buffer (50 mM, pH 6.5) and centrifuging it at 11,500× *g* for 15 min. The supernatant (2 mL) was mixed with the reaction mixture and centrifuged again at 11,500× *g* for 12 min after incubating at room temperature for 10 min. The absorbance of the final supernatant was read at 410 nm spectrophotometrically, calculated using 0.28 μM^−1^cm^−1^ as the extinction coefficient, and expressed as nmol/g FW [45].

### 4.4. Photosynthetic Pigment Contents

Photosynthetic pigments were determined using the method described by Arnon [46] and Wellburn [47], homogenizing 0.25 g of fresh leaf in 10 mL of 80% Acetone on an ice cold mortar and centrifuging it at 2000× *g* for 10 min. The supernatant was then read/observed spectrophotometrically at 663, 645, and 470 nm and the values of chlorophyll (chl) *a*, *b*, chl (*a* + *b*), and carotenoids (car) were then calculated.

### 4.5. Relative Water Content (RWC) and Proline Content

Relative water content (RWC) was measured according to Barrs and Weatherly [48]. Five randomly selected, fully developed leaves were weighed, recorded as FW, and sunk into deionized water in a petri dish for 12 h. Then, the leaves were removed from the water and excess surface water was blotted with paper towels, and the leaves were weighed and recorded as turgid weights (TW). The leaves were dried at 80 °C for 48 h for achieving DW and RWC was calculated with the following formula:

RWC (%) = [(FW-DW)/ (TW-DW)] × 100(1)

For determining proline (Pro), 0.25 g fresh leaves were homogenized in 3% sulfo-salicylic acid and centrifuged at 11,500× *g*. The supernatant was further mixed with acid ninhydrin solution (ninhydrin in glacial acetic acid mixed with 6 M phosphoric acid) and glacial acetic acid in an equal proportion and incubated at 100 °C for an hour. The mixture was then cooled and toluene was added, and the sample was mixed thoroughly for separating the Pro chromophore, read at 520 nm using a spectrophotometer and calculated by comparison with a standard curve [49].

### 4.6. Nonenzymatic Antioxidant Assay

Nonenzymatic antioxidants AsA and GSH were assayed after extracting 0.5 g of leaf sample in 3 mL 5% meta-phosphoric acid containing 1 mM Ethylenediaminetetraacetic acid (EDTA) and centrifuging it at 11,500× *g* for 12 min to remove all plant debris and to get the clear supernatant. The supernatant was neutralized using K-P buffer (0.5 M, pH 7.0) and used to measure AsA and GSH. For measuring total AsA, 0.1 M dithiothretitol was added to convert the DHA to AsA. Then, AsA (total and reduced) was assayed at 265 nm spectrophotometrically using a standard curve and DHA was calculated after subtracting reduced AsA from total AsA [14]. Glutathione was determined by enzymatic recycling and the rate of absorption change was read by a spectrophotometer at 412 nm and plotted against standard curves with known concentrations of GSH and GSSG. 2-vinylpyridine dependent removal of GSH was used to determine the GSSG content. Finally, the content of GSH was calculated by subtracting GSSG from total GSH [14]. 

### 4.7. Protein Determination and Enzyme Activity Assay

For the assaying of soluble protein and enzymatic activity, leaf samples (0.5 g) were homogenized with 1 mL of ice-cold extraction reagent containing 1 mM AsA, 50 mM K-P buffer (pH 7.0), 100 mM KCl, 5 mM β-mercaptoethanol, and 10% (*w/v*) glycerol, followed by centrifugation at 11500× *g* for 10 min, which was further used for the assay whilst maintaining a temperature 0–4 °C. 

The protein determination involved the binding of Coomassie brilliant blue dye with a soluble protein, which was thus read under 595 nm and calculated using a standard curve [50].

Lipoxygenase (LOX; EC: 1.13.11.12) activity was assayed according to Doderer et al. [51] using linoleic acid as a substrate at 234 nm spectrophotometrically. 

Superoxide dismutase (SOD; EC 1.15.1.1) activity was estimated based on the xanthine–xanthine oxidase system [52]. The reaction mixture contained K-P buffer (50 mM), NBT (2.24 mM), CAT (0.1 units), xanthine oxidase (0.1 units), and xanthine (2.36 mM), and was expressed as U min^−1^ mg^−1^ protein.

Catalase (CAT; EC: 1.11.1.6) activity was assayed following Hasanuzzaman et al. [53] by monitoring the decrease in absorbance at 240 nm, and activity was calculated using 39.4 M^−1^cm^−1^ as the extinction coefficient.

Glutathione peroxidase (GPX; EC: 1.11.1.9) activity was enumerated according to Elia et al. [54], using reaction buffer containing 100 mM K-P buffer (pH 7.0), 1 mM EDTA, 1mM sodium azide (NaN_3_), 0.12 mM NADPH, 2 mM GSH, and 1 unit of GR, using 0.6 mM H_2_O_2_ as the substrate, and the activity was expressed as nmol min^−1^mg^−1^ of protein.

Glutathione S-transferase (GST; EC: 2.5.1.18) activity was measured spectrophotometrically according to Hasanuzzaman et al. [16], using a reaction mixture containing 1.5 mM GSH and 1 mM 1-chloro-2,4-dinitrobenzene (CDNB). The increase of absorbance occurred due to the conjugation of CDNB with GSH, which was read at 340 nm for a min. The enzyme activity was calculated using the extinction coefficient of 9.6 mM^−1^cm^−1^. 

Ascorbate peroxidase (APX; EC: 1.11.1.11) activity was determined as stated by Nakano and Asada [55], where the assay mixture included K-P buffer (50 mM, pH 7.0), EDTA (0.1 mM), AsA (0.5 mM), and H_2_O_2_ (0.1 mM). The activity of APX was computed using 2.8 mM^−1^cm^−1^ as the extinction coefficient.

Monodehydroascorbate reductase (MDHAR, EC: 1.6.5.4) activity was assayed following Hossain et al. [56], using 703.4 µL of reaction mixture consisting of Tris-HCl buffer (50 mM, pH 7.5), AsA (2.5 mM), NADPH (0.2 mM), and AO (0.5 unit), and was read at 340 nm and calculated using 6.2 mM^−1^cm^−1^ as the extinction coefficient.

Dehydroascorbate reductase (EC: 1.8.5.1) activity was assayed with the mixture containing K-P buffer (50 mM, pH 7.0), GSH (2.5 mM), EDTA (0.1 mM), and DHA (0.1 mM). The activity of DHAR was observed from the increase in absorbance at 265 nm and calculated using 14 mM^−1^cm^−1^ as the extinction coefficient [55]

Glutathione reductase (EC: 1.6.4.2) activity was measured following Hasanuzzaman et al. [16] by observing the decline in absorbance at 340 nm, where the reaction mixture consisted of K-P buffer (0.1 M, pH 7.0) and EDTA (1 mM), calculated using 6.2 mM^−1^cm^−1^ as the extinction coefficient.

### 4.8. Glyoxalase Activity Assay and Methylglyoxal Content

Glyoxalase I (Gly I; EC: 4.4.1.5) activity was recorded according to Hasanuzzaman et al. [16], where the reaction mixture contained K-P buffer (100 mM, pH 7.0), magnesium sulfate (15 mM), GSH (1.7 mM), and MG (3.5 mM). After adding MG, the reaction was started and the increase in absorbance was obtained at 240 nm for 1 min. The activity of Gly I was computed using 3.37 mM-1cm-1 as the extinction coefficient.

Glyoxalase II (EC: 3.1.2.6) activity was assayed by Principato et al. [57], where 500 µL of the reaction mixture contained Tris-HCl buffer (100 mM, pH 7.2), DTNB (0.2 mM), and S-D-lactoylglutathione (SLG, 1 mM). The increase of absorbance was recorded at 412 nm spectrophotometrically and counted using 13.6 mM^−1^cm^−1^ as the extinction coefficient. 

Methylglyoxal was determined after extracting 0.25 g leaf in 5% perchloric acid and centrifuged at 11,000× *g*, decolorized using charcoal, and subsequently neutralized by Na_2_CO_3_. The neutralized supernatant was then used for the N-acetyl-l-cysteine assay for MG estimation at a wavelength of 288 nm using a standard curve [58]. 

### 4.9. Statistical Analysis

The data obtained for various morphophysiological and biochemical parameters were then subjected to statistical analysis using XLSTAT 2017 [59] software and the mean differences were separated using Fisher’s LSD test (*p* ≤ 0.05). 

## 5. Conclusions

Apart from the optimal pH, when plants were exposed to extreme pH conditions, higher H_2_O_2_ and MG production created subsequent oxidative stress, produced MDA together with enhanced LOX activity, and disturbed plant antioxidant defense and glyoxalase systems. Moreover, a reduction in water balance and photosynthetic pigments content leads to poor growth and development. However, modulating the coordinated actions of the antioxidant defense and glyoxalase system could be an important strategy in improving the performance of wheat seedlings under extreme pH-stress. In sum, our findings provide information regarding the points of damage in antioxidant defense and glyoxalase systems. Thus, these findings might further assist in selecting extreme pH (acidity or alkalinity)-tolerant genotypes. Moreover, researchers might get information for discovering suitable phytoprotectants to tinker the antioxidant defense and glyoxalase system under extreme pH-stress.

## Figures and Tables

**Table 1:** Shoot and root fresh weight, shoot and root dry weight, leaf RWC, and Pro content in leaves of wheat (*Triticum aestivum* L) seedlings under different levels of pH. Means (±SD) were calculated from three replications (*n* = 3) for each treatment. Values with different letters are significantly different at *p* ≤ 0.05 when applying Fisher’s LSD test.

Treatments	Plant Height (cm)	Root Length (cm)	Shoot FW (g plant^−1^)	Root FW (g plant^−1^)	Shoot DW (g plant^−1^)	Root DW (%)	Leaf RWC (%)	Pro (nmol g^−1^FW)
pH 4.0	10.32 ± 0.23c	5.34 ± 0.04c	0.98 ± 0.003b	0.17 ± 0.001d	0.15 ± 0.002b	0.05 ± 0.001c	82.31 ± 0.53c	5.53 ± 0.09b
pH 5.5	13.17 ± 0.37b	6.18 ± 0.08b	1.06 ± 0.056a	0.21 ± 0.002b	0.16 ± 0.002a	0.07 ± 0.002b	88.67 ± 1.23b	1.08 ± 0.04c
pH 7.0	15.33 ± 0.57a	6.52 ± 0.10a	1.12 ± 0.014a	0.24 ± 0.001a	0.16 ± 0.002a	0.07 ± 0.001a	94.31 ± 0.44a	0.30 ± 0.01d
pH 8.5	10.52 ± 0.13c	5.32 ± 0.02c	0.93 ± 0.003b	0.18 ± 0.001c	0.15 ± 0.001b	0.05 ± 0.001c	81.12 ± 0.21c	6.57 ± 0.10a

## Supplementary Materials

The following is available online at http://www.mdpi.com/2223-7747/8/1/24/s1, Figure S1: Work flow scheme of the conducted experiment.
