# Supplementary figures and images for: Unraveling Morphophysiological and Biochemical Responses of Triticum aestivum L. to Extreme pH: Coordinated Actions of Antioxidant Defense and Glyoxalase Systems
Source: Plants (Basel). 2019 Jan 18;8(1):24. doi: 10.3390/plants8010024 (PMC6359243; doi:10.3390/plants8010024)

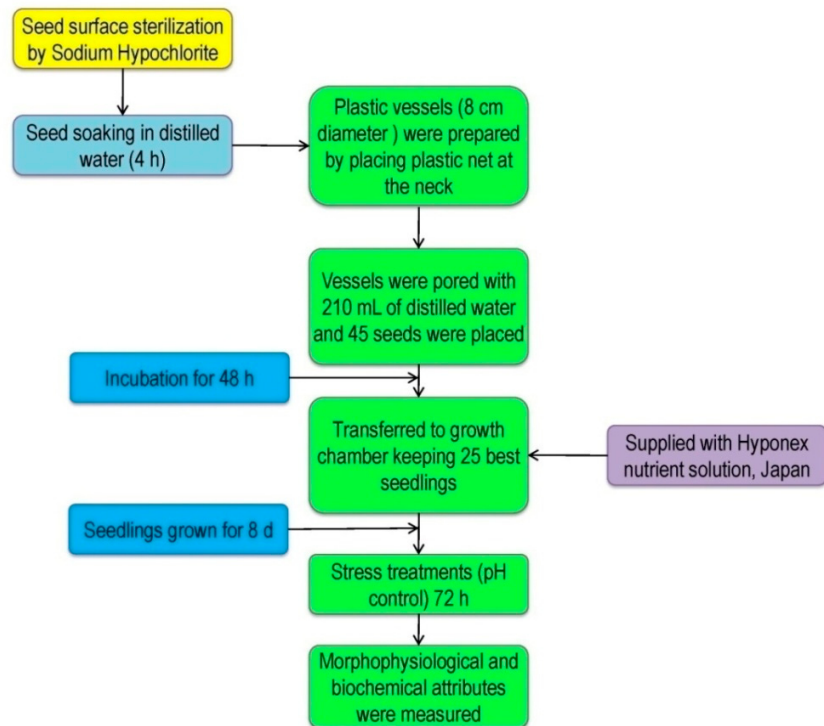

**Figure S1.** Work flow scheme of the conducted experiment.

Supplement: Supplementary file 1 [file plants-08-00024-s001.pdf]
